# Initiation of the flexirubin biosynthesis in *Chitinophaga pinensis*

**DOI:** 10.1111/1751-7915.12110

**Published:** 2014-01-28

**Authors:** Tim A Schöner, Sebastian W Fuchs, Christian Schönau, Helge B Bode

**Affiliations:** 1 Merck Stiftungsprofessur für Molekulare Biotechnologie, Fachbereich BiowissenschaftenMax-von-Laue-Str. 9, Frankfurt am Main, 60438, Germany; 2 Sanofi R&D, Industriepark HöchstFrankfurt am Main, Germany

## Abstract

Bacteria from the Bacteroidetes phylum are known producers of the chemotaxonomic relevant flexirubins. These orange pigments comprise a non-isoprenoid aryl-polyene carboxylic acid esterified with a dialkylresorcinol. Herein, we report a gene cluster from *Chitinophaga pinensis* encoding the biosynthesis of the polyene moiety and the biochemical characterization of a tyrosine ammonia-lyase and a 4-coumarate-CoA ligase responsible for the initiation of the polyene biosynthesis. Additionally, the flexirubin of *C. pinensis* was characterized by a combination of feeding experiments, high-performance liquid chromatography tandem mass spectrometry and matrix-assisted laser desorption/ionization mass spectrometry.

## Introduction

**Figure 1:**
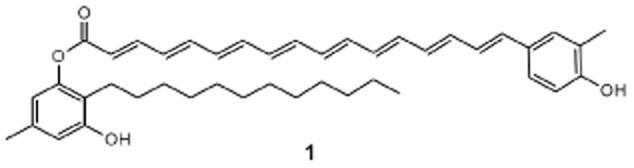
Structure of flexirubin (1) from *Chitinophaga filiformis* and *Chitinophaga pinensis.*

Since the discovery of flexirubin (**1**) (Fig. 1) in *Chitinophaga filiformis* (previously *Flexibacter elegans* Fx e1) (Reichenbach *et al*., 1974; Kämpfer *et al*., 2006), flexirubin-type pigments have been used as chemotaxonomic markers for the bacteria of the Bacteroidetes phylum (previously called the Cytophaga–Flavobacterium–Bacteroides group). Their conserved structural feature is a ω-(4-hydroxyphenyl)-polyene carboxylic acid chromophore, esterified with a 2,5-dialkylresorcinol (DAR). Known derivatives differ in methylation and/or chlorination pattern at the polyene ring and the chain lengths of the DAR alkyl chains as well as the polyene chain length (Achenbach *et al*., 1982). Feeding experiments with radioactive precursors combined with chemical degradation showed that tyrosine is the precursor of the polyene ring and that its methyl group is derived from methionine (Achenbach *et al*., 1979). As labelled acetate and malonate were also incorporated, it was proposed that fatty acid and/or polyketide biosynthesis mechanisms are involved (Fautz and Reichenbach, 1979). Whereas we could show previously that the DAR moiety is derived from a condensation of two fatty acid metabolism intermediates by DAR enzymes (Fuchs *et al*., 2013), the enzymes for the biosynthesis of the polyene moiety are still unknown. However, mutant strains of *Flavobacterium johnsoniae* UW101 revealed that the DAR biosynthesis genes from this flexirubin producer are flanked by other genes encoding proteins involved in flexirubin biosynthesis (McBride *et al*., 2009). One gene in this gene cluster encodes a putative enyzme from the aromatic amino acid lyase family. This enzyme family comprises histidine ammonia-lyases (HALs), phenylalanine ammonia-lyases (PALs) and tyrosine ammonia-lyases (TALs). HALs are common in bacteria and humans and deaminate histidine to urocanic acid (Michal, 1999). PALs convert phenylalanine to *E*-cinnamic acid (CA), whereas TALs deaminate tyrosine to 4-coumarate (4-hydroxycinnamic acid, 4CA). Whereas PAL and TAL are ubiquitous in plants and fungi, only a few examples were found in bacteria, such as *Streptomyces maritimus* PAL (Xiang and Moore, 2002), *Photorhabdus luminescens* PAL (Williams *et al*., 2005) *Rhodobacter capsulatus* TAL (Kyndt *et al*., 2002), *Rhodobacter sphaeroides* TAL (Xue *et al*., 2007), *Streptomyces* sp. Tü4128 TAL (Zhu *et al*., 2012) and *Saccharothrix espanaensis* TAL (Berner *et al*., 2006). 4CA or CA can be activated by 4-coumarate-CoA ligases (4CL) or *E*-cinnamate-CoA ligases (CCL). 4CL are common in plants where they catalyse the last reaction in the phenylpropanoid pathway and have been extensively studied in the past. Only little is known about enzymes with 4CL activity from bacteria; as to the best of our knowledge, only the enzyme ScCCL from *Streptomyces coelicolor* A3 (2) (Kaneko *et al*., 2003) and an unknown enzyme from *Pseudomonas putida* (Zenk *et al*., 1980) have been shown *in vitro* to have 4CL activity. Furthermore, hints for the presence of 4CL in bacterial biosynthetic pathways were gained in works about the biosynthesis of enterocin in *S. maritimus* (Hertweck and Moore, 2000) or the photoactive yellow protein in *R. capsulatus* (Kyndt *et al*., 2003).

Here, we describe the putative flexirubin biosynthesis gene cluster of *Chitinophaga pinensis* and the biochemical characterization of the TAL as well as the 4CL, both involved in the start of the polyene biosynthesis. Furthermore, we characterized the flexirubin from *C. pinensis* by a combination of stable isotope labelling, high-performance liquid chromatography–electrospray ionization tandem mass spectrometry (HPLC-ESI-MS) and matrix-assisted laser desorption/ionization mass spectrometry (MALDI-MS), presenting an alternative method for the detection of this pigment class in bacteria.

## Results and discussion

**Figure 2:**
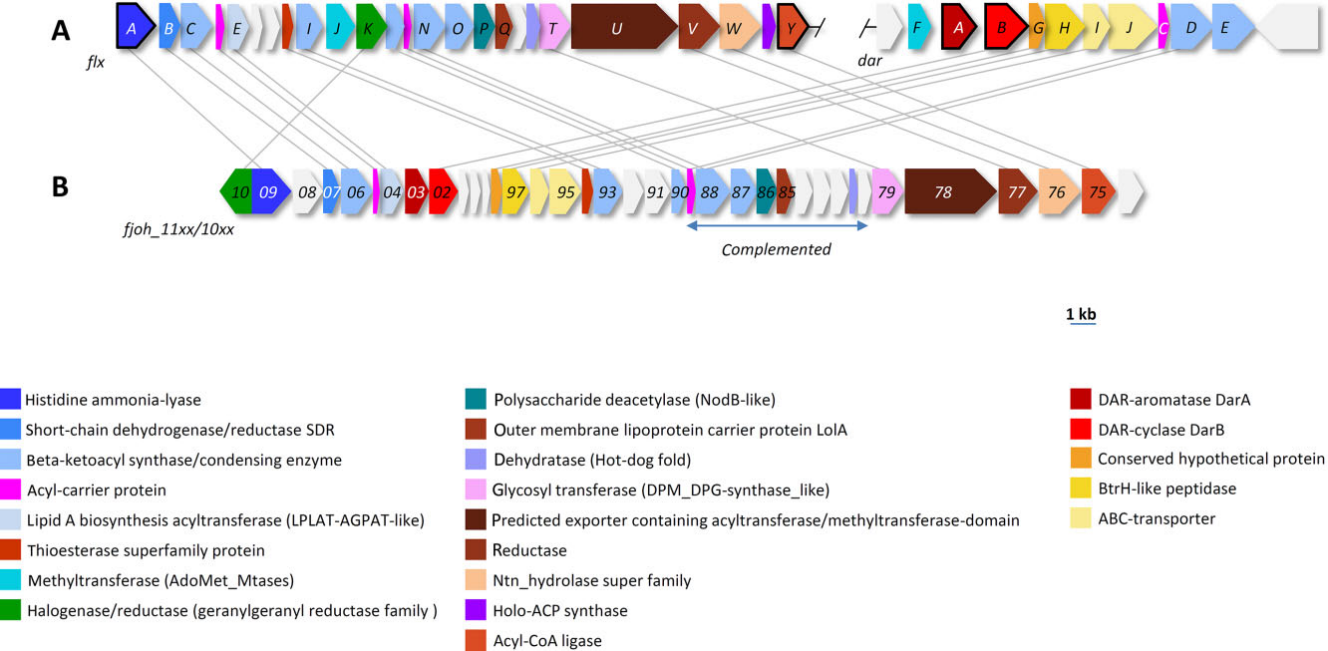
Known and proposed gene clusters for the biosynthesis of flexirubins in *Chitinophaga pinensis* DSM 2588 (A) and *Flavobacterium johnsoniae* UW101 (B). Genes encoding proteins FlxA, FlxY, DarA and DarB are shown with black frames. The blue arrow shows the region that complemented a spontaneous flexirubin-negative strain of *F. johnsoniae* (McBride *et al*., 2009). Colours depict genes with the same annotation, which are connected by grey lines if their identity was ≥ 40% in a BLAST-P analysis. All genes are scaled to the depicted scale bar.

**Figure 3:**
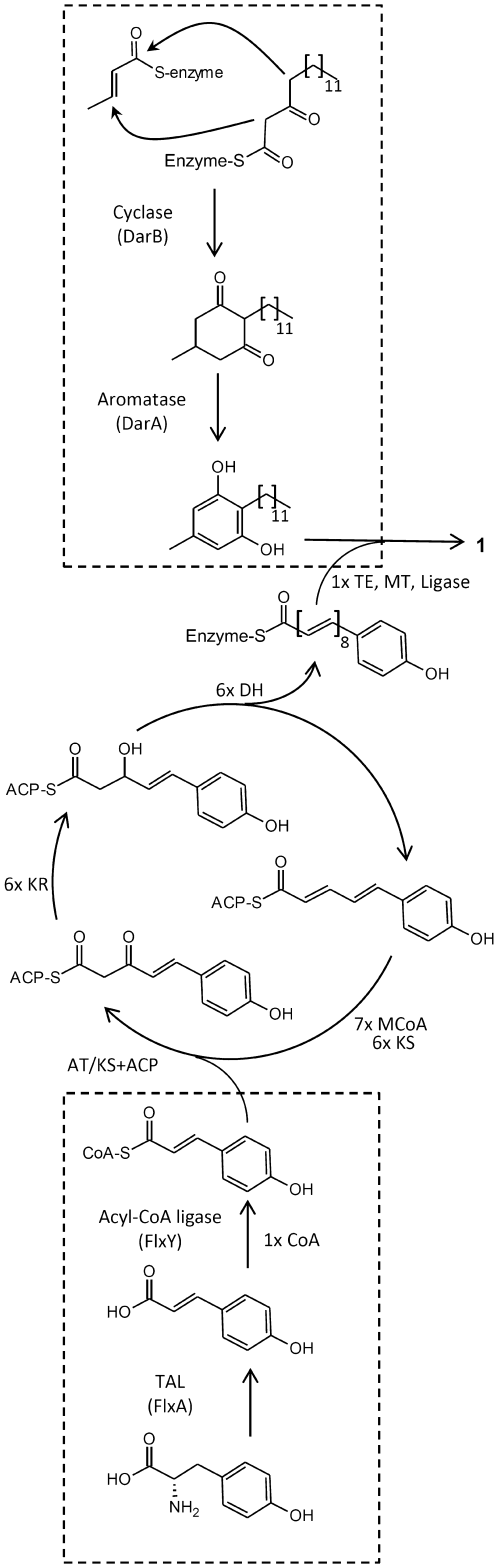
Postulated biosynthesis of flexirubin in *Chitinophaga pinensis*. Dashed frames mark reactions previously shown or reported in this work. AT: acyltransferase; KS: ketosynthase; ACP: acyl carrier protein; KR: ketoreductase; DH: dehydratase; MCoA: malonyl-CoA; TE: thioesterase; MT: methyltransferase.

We previously reported that heterologous expression of the genes *darB* (Cpin_6850) and *darA* (Cpin_6851) (Fig. 2A) from *C. pinensis* led to the production of a DAR, which is identical to the DAR moiety in flexirubin **1** from *C. filiformes* (Fuchs *et al*., 2013). Additionally, these genes have also been found in *F. johnsoniae* UW101 where mutation of *darB* (Fjoh_1102) (Fig. 2B) resulted in a flexirubin-negative phenotype (McBride *et al*., 2009). In *F. johnsoniae*, *darA* and *darB* are part of a 37 kbp gene cluster and a spontaneous flexirubin-negative mutant was complemented by a plasmid carrying part of this cluster (Fjoh_1078 to Fjoh_1089) (Fig. 2B), which proved that additional genes from this cluster are involved in flexirubin biosynthesis (McBride *et al*., 2009). However, no further characterization of this gene cluster was reported. STRING analysis (Franceschini *et al*., 2013) of the *C. pinensis* genome with the *F. johnsoniae* flexirubin gene cluster revealed a homologous gene cluster spanning Cpin_1853 (*flxA*) to Cpin_1877 (*flxY*) and the already reported DAR gene cluster (Fig. 2A and Tables S4 and S5). In the gene cluster, several putative β-ketoacyl synthases, reductases, dehydratases and thioesterases are encoded, suggesting a type II fatty acid synthase-like biosynthesis of the polyene moiety (Fig. 3). As the polyene-aryl and the adjacent double bond are derived from tyrosine (Achenbach *et al*., 1979), we speculated that the predicted HAL FlxA (Cpin_1853) encoded in the biosynthesis gene cluster, in fact, catalyses the deamination of l-tyrosine to 4CA, which might then be activated for the polyketide synthase (PKS) machinery by adenylation through the putative acyl-CoA ligase FlxY (Cpin_1877) (Fig. 3). 4-Coumaroyl-CoA may then be used as precursor for a fatty acid-like biosynthesis of the polyene by chain elongation through the putative β-ketoacyl synthases (FlxC, FlxI, FlxL, FlxN, FlxO) followed by reduction of the β-keto function by FlxB or FlxV and generation of a double bond by a dehydratase (FlxF, FlxS). The aryl-octaene moiety may then be connected to the DAR by a ligase-like enzyme (FlxW). Flexirubin is found in the outer membrane (Irschik and Reichenbach, 1978). Thus, *flxP*-*flxU*, encoding a putative polysaccharide deacetylase, a phospholipid/glycerol acyltransferase, an outer membrane lipoprotein carrier LolA, a glycosyltransferase and a predicted exporter may be involved in the export of the pigment by yet unknown mechanisms.

### Tyrosine ammonia-lyase activity

**Figure 4:**
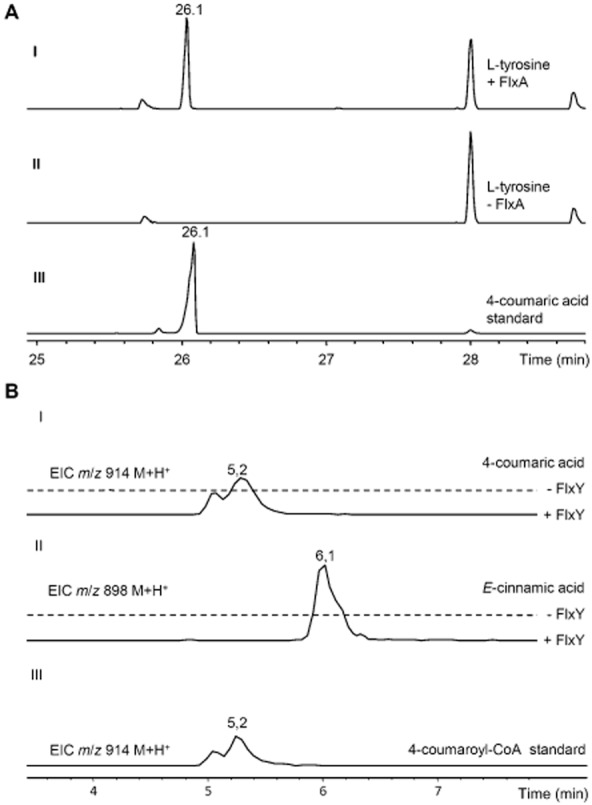
Analysis of assays with purified enzymes from the flexirubin biosynthesis.
GC-MS analysis (TIC, positive mode) of l-tyrosine containing assays with (I) or without FlxA (II) and of 4-coumaric acid standard (III).HPLC-MS analysis of assays containing 4-coumaric acid (I) or *E*-cinnamic acid (II) with (solid line) or without (dashed line) FlxY and of 4-coumaroyl-CoA standard (III).

Because two motives were identified in the past that might allow the prediction of the substrate specificity of aromatic amino acid ammonia-lyases, an alignment of the FlxA primary sequence with those of eight ammonia-lyases with known substrate specificity was performed (Fig. S1). However, the amino acids at motive 1 (Watts *et al*., 2006) were similar to those of HAL enzymes and not TAL/PAL, whereas at motive 2 (Berner *et al*., 2006), the conserved residue from TAL or PAL enzymes was found. To get experimental evidence whether FlxA has ammonia-lyase activity, it was heterologously produced in *Escherichia coli*. Subsequent purification of FlxA (Fig. S2) allowed its incubation with putative substrates and the enzyme assays were analysed by gas chromatography–mass spectrometry (GC-MS). In assays containing FlxA and l-tyrosine, 4CA was detected (Fig. 4A and Fig. S3A), whereas l-phenylalanine was deaminated to CA (Fig. S3B). No product formation was detectable with l-histidine, l-isoleucine and l-tryptophane as substrates (data not shown). The same results were obtained in a photometric assay (Fig. S4A). To confirm the specificity of FlxA, photometric assays with l-tyrosine or l-phenylalanine as substrate were optimized towards pH and temperature (Fig. S4B). The linear plots of the Michaelis–Menten kinetics (Fig. S5A–D) revealed *k*_cat_/*K*_M_ values of 7728 M^−1^ s^−1^ for l-tyrosine and 7.7 M^−1^ s^−1^ for l-phenylalanine. Although other bacterial TALs showed a 150-to 300-fold higher catalytic activity with l-tyrosine (Watts *et al*., 2006), FlxA showed a 1010-fold higher specificity towards l-tyrosine against l-phenylalanine, supporting its proposed function in the flexirubin biosynthesis.

### CoA ligase activity

As the polyene-aryl and the adjacent double bond from flexirubin are derived from tyrosine (Achenbach *et al*., 1979), we concluded that the 4CA produced by FlxA may be activated by the putative acyl-CoA ligase FlxY to serve as substrate for the following PKS biosynthesis of the polyene. An alignment of the primary sequence from FlxY with those from ScCCL from *S. coelicolor* and three 4CL isoforms from *Arabidopsis thaliana* is shown in Fig. S6. The alignment shows that, in contrast to ScCCL (44% identity to At4Cl2), FlxY is not closely related to plant 4CLs (< 12% identity to At4CL1–3) and several residues in reported conserved domains differ from those of plant 4CLs (Schneider *et al*., 2003) or ScCCL (Kaneko *et al*., 2003), preventing an accurate prediction of the catalysed reaction. Therefore, we tested the acyl-CoA ligase activity of FlxY *in vitro*. After heterologous overexpression in *E. coli* and subsequent purification (Fig. S2), its ability to perform the postulated reaction was tested in enzyme assays. HPLC-ESI-MS analysis of the enzyme assay with 4CA as substrate revealed the formation of a compound with *m*/*z* 914 [M + H]^+^ (Fig. 4BI and Fig. S7AIII) and the same retention time and MS^2^ as 4-coumaroyl-CoA (Fig. 4BIII and Fig. S7AII), whereas in the presence of CA, a compound with *m*/*z* 898 [M + H]^+^ was formed that fragmented in MS^2^ experiments very similar to 4-coumaroyl-CoA but with −Δ16 Da mass shifts as expected for *E*-cinnamoyl-CoA (Fig. 4BII and Fig. S7AIV). Furthermore, *E*-3-indoleacrylic acid, indole-3-propionic acid, 3-chlorocinnamic acid and 3-phenylpropionic acid were also accepted as substrates, but other carboxylic acids were not (Fig. S8 and Table S3). To test the substrate specificity of FlxY, a coupled enzyme assay was used and optimized for product formation at different pH and temperatures (Fig. S9A and B). The Michaelis–Menten kinetics with 4CA and CA were determined and the linear plots (Fig. S9C–F) showed *k*_cat_/*K*_M_ values of 52977 M^−1^ s^−1^ for 4CA and 437 M^−1^ s^−1^ for CA, giving a 121-fold higher catalytic activity with 4CA. Values for the only two other biochemical characterized bacterial enzymes with 4CL activity are given in Table S6 (Zenk *et al*., 1980; Kaneko *et al*., 2003), showing that FlxY is the bacterial enzyme with the best 4CL activity reported to date.

### Flexirubin structure elucidation

**Figure 5:**
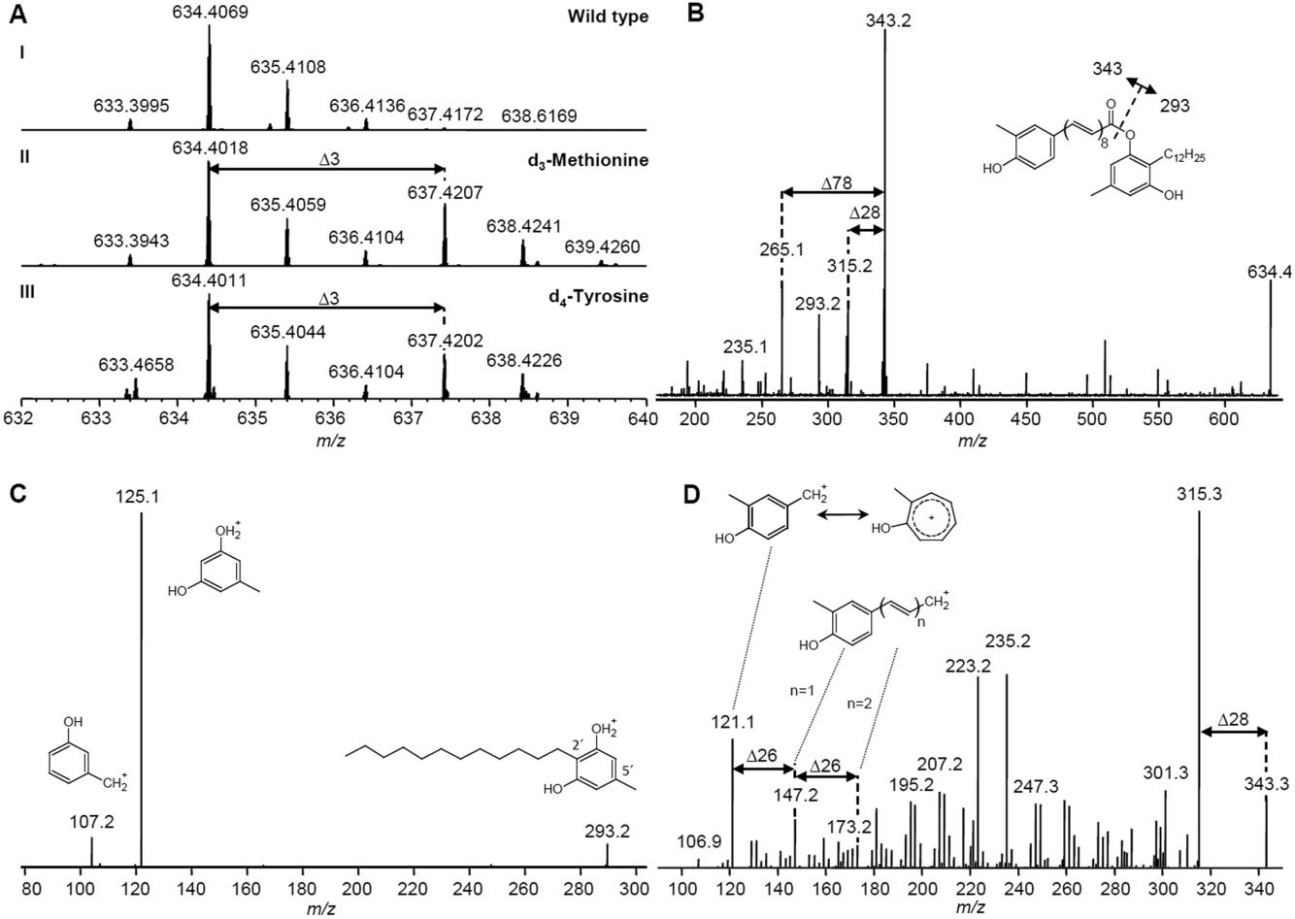
Mass spectra of flexirubin 1 from *C. pinensis*.
MALDI-orbitrap mass spectra of wild-type 1 (I) and feeding experiments with d_3_-methionine (II) and d_4_-tyrosine (III) are shown.MALDI-iontrap-MS^2^ of *m/z* 634.4 [M]^+·^. MALDI-iontrap-MS^3^ of *m/z* 293.2 (C) and *m/z* 343.3 (D) with *m/z* 634.4 [M]^+^· as precursor.

As *C. pinensis* is a close relative of *C. filiformes* in the Bacteroidetes phylum (Kämpfer *et al*., 2006), we were interested whether both strains also produce the same flexirubin derivative. Crude acetone extracts of *C. pinensis* cultures were yellow, and a reversible colour shift upon treatment with alkali (Fig. S10A) suggested the presence of flexirubin as described (Fautz and Reichenbach, 1980). Subsequent purification of the yellow compound and MALDI-MS analysis led to mass of *m/z* 634.40217 [M]^+^· and a sum formula of C_43_H_54_O_4_ (calculated *m/z* 634.40166 [M]^+^·; Δppm 0.8). The formation of a radical cation may be explained due to direct absorption of the laser light by the polyene instead of matrix-mediated ionization mechanisms. HPLC-UV-ESI-MS (Fig. S10B) confirmed that this compound also absorbs light at 420 nm, a characteristic of flexirubins. When *C. pinensis* was grown in the presence of l-[methyl-^2^H_3_]methionine, an additional signal with a mass increase of 3 Da was detected in the coloured fraction, indicating the incorporation of a methionine-derived methyl group (Fig. 5AIII). Similarly, a +3 Da mass shift was also observed when L-[ring-^2^H_4_]tyrosine was added to the culture (Fig. 5AIII), indicating its incorporation and the loss of one deuterium at the polyene ring, as expected for a methylation during the biosynthesis. Fragmentation of *m/z* 634.4 [M]^+^· by MALDI-MS led to four main fragments (Fig. 5B), already reported for fragmentation of **1** with EI-MS (Achenbach and Kohl, 1979), with *m/z* 343 and *m/z* 293 reflecting an ester cleavage, *m/*z 315.2 as loss of CO (Δ28 Da) and *m/z* 265.1 as loss of benzene (Δ78 Da) from *m/z* 343 respectively. MS^3^ of *m/z* 293.2 resulted in a mass spectrum identical to the heterologously produced DAR (Fuchs *et al*., 2013) (Fig. 5C). The detected fragments can be explained by the loss of substituents at the aromatic ring (Fig. 5C). MS^3^ of *m/z* 343.2 gave a complex fragmentation pattern with a key fragment of *m/z* 121.1 (rearrangement to the tropylium cation is expected) and mass shifts of 26 and 28 Da (Fig. 5D), which turned out to be a characteristic of flexirubin polyene fragments. MS^3^ of *m/*z 315.2 and *m/z* 265.1 showed similar fragments and fragmentation patterns and reflect shorter polyene fragments (Fig. S10D and E respectively). When *m/z* 633.5 [M − H]^−^ was fragmented by HPLC-ESI-MS^2^, *m/z* 341 and *m/z* 291 could be detected (Fig. S10C). In addition, analytical fragments with 39 and 65 Da mass shifts to *m/z* 291 were detected. To confirm the assigned fragments, MALDI-MS^2^ analysis of the labelled *C. pinensis* pigments was performed. MS^2^ analysis of *m/z* 637 [M]^+^· observed in both labelling experiments (l-[methyl-^2^H_3_]methionine or L-[ring-^2^H_4_]tyrosine) showed a mass increase of 3 Da compared to the previously assigned polyene fragments *m/z* 343, *m/z* 315 and *m/z* 265, but no mass shift for the DAR fragment *m/z* 293 (Fig. S10F, left side). Subsequent MS^3^ of the labelled polyene fragments *m/z* 346 from both feeding experiments allowed the detection of the label at the expected key fragment *m/z* 124 (Fig. S10F, right side).

Therefore, from the colour of the isolated compound, positive KOH test result, incorporation of labelled precursors, and fragmentation in MALDI-MS and ESI-MS, the structure of **1** from *C. pinensis* is most likely similar to the reported flexirubin from *C. filiformes* (Achenbach *et al*., 1976). A remaining task would be to elucidate by means of nuclear magnetic resonance or chemical degradation if the methylation is indeed in *ortho*-position to the hydroxyl function of the polyene aryl, which is so far the only observed substitution pattern for flexirubins. As this is a time-consuming and challenging process for flexirubins (Fautz and Reichenbach, 1980), it would only be justified if a new derivative is expected. Nevertheless, most of the structure elucidation of **1** has been achieved without radioactive labelling and circumventing large-scale fermentations as it has been necessary before (Reichenbach *et al*., 1974; Achenbach and Kohl, 1978).

## Conclusions

In summary, we showed that the genome of *C. pinensis* contains a flexirubin biosynthesis gene cluster, in which *flxA* encodes a TAL and *flxY* encodes a CoA ligase with specificity for 4CA. Only a few bacterial enzymes with PAL or TAL activity were reported before, whereas HAL are widely distributed. Due to the fact that, actually from sequence information alone, the substrate specificity of these enzymes cannot be predicted, the distribution of PAL and TAL enzymes in the Bacteroidetes phylum and in bacteria in general is still unknown. Even less is known about enzymes with 4CL activity from bacteria and FlxY shows a low similarity to the well-known plant 4CL enzymes. The herein reported enzymes from *C. pinensis* may therefore become already characterized examples for future studies targeting the mechanisms that confer their substrate specificity and may help to develop solid predictions based upon sequence alignments. Additionally, the first two steps in the polyene biosynthesis of flexirubins were elucidated, which have been unknown before. Furthermore, the identification of flexirubin with nowadays widespread and established HPLC-ESI-MS and MALDI-MS methods was reported and might lead to the identification of additional flexirubin producers. Its column chromatography is simple because even traces of pigment can be visually detected and less than 1 μL of enriched fraction is needed for a complete MALDI-MS^n^ analysis, enabling the characterization of flexirubins from newly described bacteria producing this chemotaxonomic marker.

## Experimental procedures

### Cultivation of strains

All strains (Table S1) were cultivated on solid Luria–Bertani (LB) medium (5 g l^–1^ of yeast extract, 10 g l^–1^ tryptone and 5 g l^–1^ NaCl) or in liquid LB medium on a rotary shaker at 200 r.p.m. For feeding experiments and extraction of chromosomal DNA, *C. pinensis* DSM 2588 was cultivated at 30°C and *E. coli* strains at 37°C. Where appropriate, kanamycin (50 μg ml^–1^) was added to the medium.

### Molecular–biological methods

Molecular–biological experiments were performed according to standard procedures (Sambrook *et al*., 1989). Polymerase chain reactions (PCRs) were performed with the Phusion high-fidelity polymerase (Thermo Fisher Scientific, Waltham, MA) according to the manufacturer's instructions and with oligonucleotides (Table S2) from Sigma-Aldrich (St. Louis, MO, USA). DNA extraction from agarose gels was performed with the GeneJet™ Gel Extraction Kit (Thermo Fisher Scientific) and plasmid isolations with the GeneJet Plasmid Extraction Kit (Thermo Fisher Scientific).

### Construction of plasmids for heterologous expression of FlxA and FlxY

The genes *cpin_1853* (*flxA*) and *cpin_1877* (*flxY*) were amplified by PCR from genomic DNA isolated from *C. pinensis*. The resulting products, 1853fragment and 1877fragment, were digested with the restriction endonucleases BamHI/XhoI and BamHI/HindIII, respectively, and ligated into the similarly treated pCOLADuet-1, followed by transformation of *E. coli* DH10B cells. The plasmids pCOLA-1853 and pCOLA-1877 were isolated and the inserts were sequenced at the SeqIT GmbH (Kaiserslautern, Germany).

### Overproduction and purification of FlxA and FlxY

*Escherichia coli* BL21 (DE3) cells were transformed with pCOLA-1853 or pCOLA-1877, resulting in strain CS1853 and CS1877 respectively. Additionally, 200 ml of LB medium was inoculated 1:100 from an overnight culture of CS1853 or CS1877 and grown to a OD_600_ of 0.6–0.8 at 37°C. Isopropyl-β-d-1-thiogalactopyranoside was added to a final concentration of 0.1 mM and cells were cultivated for 16 h at 18°C. After centrifugation, the cell pellets were frozen at –20°C. Cells were lysed according to the freeze-thaw method, by addition of a lysation buffer [500 mM NaCl, 20 mM imidazole, 20 mM tris(hydroxymethyl)aminomethane (Tris) (pH 7.5)], which additionally contained 0.005 volume of protease inhibitor cocktail set (Calbiochem, Merck, Darmstadt, Germany), lysozyme (10 kU ml^–1^) and benzonase (2.5 U ml^–1^). The soluble fractions were filtered through 0.6 μM syringe filters and applied to a ÄKTAexplorer™ System (GE Healthcare, Fairfield, CT, USA) equipped with a HisTrap™ HP 1 ml affinity chromatography column. Binding and elution buffer contained 500 mM NaCl, 20 mM imidazole and 20 mM Tris (pH 7.5), and 500 mM NaCl, 500 mM imidazole and 20 mM Tris (pH 7.5) respectively. Eluted fractions were analysed by sodium dodecyl sulfate–polyacrylamide gel electrophoresis (SDS-PAGE) and protein concentrations were determined with a Nanovue Plus Spectrophotometer (GE Healthcare). The His_6_-tagged proteins were stored at −80°C in storage buffer [100 mM NaCl, 50 mM Tris (pH 7.5), 1 mM dithiothreitol, 1 mM ethylenediaminetetraacetic acid and 10% glycerol].

### FlxA *in vitro* assays

Activity of FlxA with l-histidine, l-tyrosine, l-phenylalanine, l-isoleucine and l-tryptophan was tested in 1 ml of assays [1 μM FlxA, 1 mM l-amino acid and 50 mM Tris (pH 8.0)]. After incubation (12 h, 25°C), assay products were extracted twice with 1 volume of ethylacetate, dried and redissolved in 50 μl of CHCl_3_ followed by derivatization (55°C, 2 h) with 25 μl of *N*-methyl-*N*-trimethylsilyl-trifluoroacetamide. Reaction products were detected on a 7890A gas chromatograph (Agilent, Santa Clara, CA, USA) equipped with a 5975C mass selective detector (Agilent). A DB5ht column (30 m × 250 μm × 0.1 μM; Agilent) was used for the separation of the analytes, with helium as the carrier gas (1 ml min^–1^). The method parameters were as follows: injection volume: 2 μl; inlet temperature: 275°C; injection mode pulsed split (10:1); oven temperature programme: starting temperature 2 min at 130°C, then 5°C min^–1^ to 240°C followed by an increase to 300°C with a rate of 30°C min^–1^ or inlet temperature: 250°C; injection mode splitless; oven temperature programme: starting temperature 5 min at 70°C, then 5°C min^–1^ to 300°C. Ionization of the analyte molecules was carried out by electron impact ionization at 70 eV. For identification of the products, ‘Automated Mass Deconvolution and Identification Software’ (AMDIS) version 2.64 in combination with the NIST library was used.

To determine the substrate specificity of FlxA, photometric assays were performed in a 96-well plate using l-phenylalanine, l-tyrosine and l-histidine as substrates. Formation of the reaction products was measured as the increase of the absorption at 275 nm (*E*-cinnamic acid), 310 nm (4-coumaric acid) and 277 nm (urocanic acid) with a SpectraMax M5 Photometer (Molecular Devices, Sunnyvale, CA, USA). The pH and temperature optima were determined in 100 μl endpoint assays containing 1 μM FlxA, 50 μM l-tyrosine or 20 mM l-phenylalanine with 50 mM Tris (pH 6–10) or 50 mM Na_2_B_4_O_7_ (pH 10–12) at 45°C, 40 min for pH optima and 50 mM Tris (pH 10) at 25–60°C, 1 h for temperature optima. Michaelis–Menten kinetics were measured in photometric assays as triplicate [1 μM FlxA, 50 mM Tris (pH 10), 45°C, 5 min, 5–1000 μM l-tyrosine or 4–100 mM l-phenylalanine]. To calculate product concentrations, calibration curves with 4-coumaric acid (5–500 μM) and *E*-cinnamic acid (10–2000 μM) were obtained. The measured data for both substrates were plotted as Michaelis–Menten kinetic and its derivations, Lineweaver–Burk and Hanes–Woolf, were used to calculate *K*_M_, *v*_max_, *k*_cat_ and *k*_cat_/*K*_M_.

### FlxY *in vitro* assays

Formation of *E*-cinnamoyl-CoA and *p*-coumaroyl-CoA by FlxY was analysed by HPLC-MS as described below. Therefore, enzyme assays [1 mM ATP, 1 mM CoA, 1 mM MgCl_2_, 50 mM Tris (pH 7.5), 37°C, 1 h] were performed. Depending upon the experiment, FlxY (1 μM), *E*-cinnamic acid (1 mM) and 4-coumaric acid (1 mM) were added. In addition, formation of CoA derivatives with other acids was tested. Enzyme assays [9.4 nM FlxY, 4.6 mM diverse acids, 1.4 mM CoA, 2.8 mM ATP, 5 mM MgCl_2_, 200 mM NaCl, 50 mM Tris (pH 7.5)] were incubated overnight at 37°C, stopped by the addition of formic acid (final concentration 4.6%) and analysed by HPLC-MS and MALDI-MS. For determination of the substrate specificity of FlxY, the EnzChek Pyrophosphate Assay Kit (Life Technologies, Carlsbad, CA, USA) was used. The pH optimum was determined between pH 6.5 and pH 8.5 and with an endpoint assay (1 μM FlxY, 1 mM 4-coumaric acid, 1 mM CoA, 1 mM ATP, 1 mM MgCl_2_, 50 mM Tris, 37°C, 1 min). To test the temperature dependence, the endpoint assay was performed between 25°C and 55°C at a pH of 7.5. Michaelis–Menten kinetics were measured as quadruplicate assays [0.5 μM FlxY, 10–750 μM 4-coumaric acid or 50–7000 μM *E*-cinnamic acid, 1 mM CoA, 1 mM ATP, 1 mM MgCl_2_, 50 mM Tris (pH 7.5), 55°C, 2 min]. *K*_M_, *v*_max_, *k*_cat_ and *k*_cat_/*K*_M_ were calculated after plotting the data as Lineweaver–Burk and Hanes–Woolf plots. 4-Coumaroyl-CoA standard was obtained from MicroCombiChem (Wiesbaden, Germany).

### Feeding experiments

Four hundred millilitres of liquid LB medium were inoculated 1:100 from an overnight culture of *C. pinensis*. After 3 h, l-[methyl-^2^H_3_]methionine or l-[ring-^2^H_4_]tyrosine were fed in 1 mM portions to the cultures. Feeding was repeated after 12, 24 and 48 h, resulting in a final concentration of 4 mM. As control, a culture without feeding was cultivated at the same time. Cultures were harvested 12 h after the last feeding by centrifugation at 10,000 r.p.m.

### Extraction and isolation of compounds

Pelleted cells of liquid cultures were shaken for 30 min with 50 mL of acetone in the dark, followed by filtration to remove cell debris. The extracts were dried under reduced pressure. The raw extracts were fractionated using silica gel columns with hexane/ethylacetate (4:1; 1% acetic acid). Coloured fractions were dried under reduced pressure. All samples were stored dry at −20°C in the dark and solved in 10–50 μl of acetonitrile (ACN) before further analysis.

### HPLC-MS

HPLC-ESI-MS analysis was performed with a Dionex UltiMate 3000 system coupled to a Bruker AmaZon X mass spectrometer and an Acquity UPLC BEH C18 1.7 μm RP column (Waters, Milford, MA, USA) as described previously (Reimer *et al*., 2011).

### MALDI-MS

For MALDI-analysis, samples were mixed 1:2 with 1 μl of a 20 mM 4-chloro-α-cyanocinnamic acid in 70% ACN with 0.1% trifluoroacetic acid, spotted onto a polished stainless steel target and air-dried. MALDI-MS^n^ analysis was performed with a MALDI LTQ Orbitrap XL (Thermo Fisher Scientific) equipped with a nitrogen laser at 337 nm, as described previously (Fuchs *et al*., 2012). Spectra were analysed and possible sum formulas were calculated using Qual Browser (version 2.0.7; Thermo Fisher Scientific).

### Genome mining

For identification of *DarAB* gene clusters, the primary structures of the DarAB homologues from *C. pinensis* DSM 2588 were used for a BLAST-P identification (protein-protein-BLAST) of homologues in *F. johnsoniae* UW101. The reported biosynthesis gene cluster from *F. johnsoniae* UW101 (McBride *et al*., 2009) was used in a STRING analysis (version 9.0) (Franceschini *et al*., 2013) for the identification of the putative flexirubin polyene cluster in *C. pinensis*. Gene colours in Fig. 2 are based upon NCBI annotation or domain-guided annotations from BLAST-P with primary sequences of *C. pinensis* genes as template (see Table S4 and S5). Grey connections between genes depict an identity ≥ 40%.

## Supporting information

**Fig. S1.** ClustalW multiple alignment of primary sequences of FlxA from *C. pinensis* with those of histidine ammonialyases (HALs), phenylalanine ammonia-lyases (PALs) and tyrosine ammonia-lyases (TALs) with known substrate specificity. Sequences used for the alignment were abbreviated as follows (with GenBank accession numbers in parentheses): HAL *P. putida*, HAL *Pseudomonas putida* (P21310); HAL *S. griseus*, HAL *Streptomyces griseus* (AAA26769); PAL *P. crispum*, PAL *Petroselinum crispum* (P24481); PAL *P. luminescens*, PAL *Photorhabdus luminescens* sp. *laumondii* TT01 (NP_929491); PAL *S. maritimus*, PAL *Streptomyces maritimus* (AAF81735); TAL *C. pinensis*, TAL *Chitinophaga pinensis* (YP_003121550); TAL *R. sphaeroides*, TAL *Rhodobacter sphaeroides* 2.4.1 (YP_355075); TAL *S. espanaensis*, TAL *Saccharothrix* *espanaensis* (ABC88669); TAL *S.* sp. Tü 4128, TAL *Streptomyces* sp. Tü 4128 (AEV23249). The selectivity switch reported by Watts *et al*. (2006) is boxed in motive 1. The residue at the first position (here residue 140) varied between Phe (F) and His (H) in PAL sequences, whereas TAL or PAL with TAL activity have a His (H) at the first position. Furthermore, a second conserved residue was reported (Berner *et al*., 2006), which is boxed in motive 2 (here residue 507) and was always Glu (E) for HAL and Gln (Q) for TAL or PAL. The alignment was performed using Geneious 6.1.7 ClustalW default settings. Coloured residues indicate a similarity ≥ 75%. **Fig. S2.** SDS-PAGE analysis of purified proteins. Left side: Purified FlxA (expected size 58 kDa). Right side: Purified FlxY (expected size 49 kDa). Marker: PageRuler™ Unstained protein ladder (Fermentas). **Fig. S3.** GC-MS analysis of FlxA enzyme assays. A. Mass spectrum of the product observed after incubation of FlxA with L-tyrosine (I) and 4-coumaric acid standard (II). B. Chromatogram of an assay containing FlxA and L-phenylalanine (I), the mass spectrum of the product at 6.1 min from the above chromatogram (II) and the mass spectrum of *E-cinnamic* acid standard (III). **Fig. S4.** Photometric FlxA enzyme assays. A. Endpoint assay detecting the enzymatic conversion of L-tyrosine (blue), L-phenylalanine (red) and L-histidine (green) by measuring the absorption increase of 4-coumaric acid (310 nm), *E*-cinnamic acid (275 nm) or urocanic acid (277 nm) respectively. B. pH dependence of FlxA activity with L-tyrosine (I) and L-phenylalanine (II). Temperature dependence of FlxA activity with K-tyrosine (III) and L-phenylalanine (IV). TAL: tyrosine ammonia-lyase; PAL: phenylalanine ammonia-lyase; HAL: histidine ammonia-lyase. **Fig. S5.** Michaelis–Menten kinetics of FlxA. Substrate saturation plots of FlxA with L-tyrosine (A) and L-phenylalanine (B) and their respective linearization as Lineweaver–Burk (C, D) or Hanes–Woolf plots(E, D). TAL: tyrosine ammonia-lyase; PAL: phenylalanine ammonia-lyase. **Fig. S6.** ClustalW multiple alignment of primary sequences of FlxY from *Chitinophaga pinensis* with three 4-coumarate-CoA ligases (4CL) from *Arabidopsis thaliana* and the *E*-cinnamate-CoA ligase (CCL) ScCCL from *Streptomyces* *coelicolor* A3(2). Sequences used for the alignment were abbreviated as follows (with GenBank accession numbers in parentheses): At4CL1, *A. thaliana* 4CL1 (AAA82888.1); At4CL2, *A. thaliana* 4CL2 (AF106086_1); At4CL3, *A. thaliana* 4CL3 (AF106088_1); ScCCL, *S. coelicolor* A3 (2) CCL (CAB95894); Cp4CL, *C. pinensis* 4CL (YP_003121574.1). Two conserved peptide boxes from 4CL are boxed in red and nine residues that are suggested to form the At4CL substrate binding pocket are marked by red triangles (Kaneko *et al*., 2003). The alignment was performed using Geneious 6.1.7 ClustalW default settings. Coloured residues indicate a similarity ≥ 75%. **Fig. S7.** HPLC-MS analysis of enzyme assays with FlxY. A. Mass spectra of CoA (I), 4-coumaroyl-CoA standard (II) and products detected in enzyme assays containing FlxY and 4-coumaric acid (III) or *E*-cinnamic acid (IV) as substrates. B. Theoretical fragmentation tree explaining the above mass spectra with randomly positioned charges. **Fig. S8.** HPLC-MS analysis of the substrate specificity of FlxY. The structures of the substrates, the EIC from CoA (green) and the EIC of expected substrate-CoA thioesters (blue) are shown. **Fig. S9.** Results of enzyme assays with FlxY. pH (A) and temperature optimum (B) of the coupled enzyme assay. Substrate saturation plots of FlxY with 4-coumaric acid (C) and *E*-cinnamic acid (D) and their respective linearization as Lineweaver–Burk (E, F) or Hanes–Woolf plots (G, H). **Fig. S10.** KOH test, mass spectra and HPLC traces from *C. pinensis* flexirubin **1**. A. Crude acetone extract of *C. pinensis* culture (left) showing the reversible colour shift after addition of base (middle) or base and acid (right). B. HPLC-MS-chromatograms of *C. pinensis* extract before (upper trace) and after fractionation by column chromatography (lower trace). UV at 420 nm (blue line) and an EIC m/z 633.5 [M − H+] are shown. The occurrence of an additional signal after purification may be explained by isomerization. Chromatograms are drawn to the same scale. C. HPLC-ESI-MS2 of 1 *m/z* 633.5 [M − H+]. MALDI-iontrap-MS3 spectra of *m/z* 315.2 (D) and *m/z* 265.3 (E) with *m/z* 634.4 [M]+ as precursor. F. On the left side, MALDI-iontrap-MS2 of wild-type **1** *m/z* 634.4 [M]+ (I) and *m*/*z* 637.4 [M]+ from feeding experiments with d3-methionine (II) and d4-tyrosine (III). The resulting fragments *m/z* 343.2 for wild type and *m*/*z* 346.2 or *m/z* 346.3 for the feeding experiments were further fragmented and the corresponding MS3 mass spectra are depicted on the right side. **Table S1.** Strains and plasmids used in this work. **Table S2.** Primers and PCR products used in this study. **Table S3.** High-resolution MALDI-orbitrap-MS of CoAthioesters from FlxY substrate assays. **Table S4.** Predicted gene clusters for flexirubin biosynthesis in *C. pinensis.* Domain-guided annotation is based on conserved domains detected by BLAST-P of *C. pinensis* DSM 2588 primary sequences against the genome of *F. johnsoniae* UW101. **Table S5.** Gene cluster for flexirubin biosynthesis in *F. johnsoniae* UW101. Domain-guided annotation is based on conserved domains detected by BLAST-P. **Table S6.** Comparison of the enzymatic properties of bacterial 4CLs (4-coumarate-CoA ligase) and CCLs (cinnamate-CoA ligase). Values were obtained from assays with 4-coumarate (4CA) and *E*-cinnamate (CA) as substrates.
