# Supplementary material for: Initiation of the flexirubin biosynthesis in Chitinophaga pinensis
Source: Microb Biotechnol. 2014 Jan 28;7(3):232–41. doi: 10.1111/1751-7915.12110 (PMC3992019; doi:10.1111/1751-7915.12110)
Supplement: Supplementary file 1 — Fig. S1. ClustalW multiple alignment of primary sequences of FlxA from C. pinensis with those of histidine ammonialyases (HALs), phenylalanine ammonia-lyases (PALs) and tyrosine ammonia-lyases (TALs) with known substrate specificity. Sequences used for the alignment were abbreviated as follows (with GenBank accession numbers in parentheses): HAL P. putida, HAL Pseudomonas putida (P21310); HAL S. griseus, HAL Streptomyces griseus (AAA26769); PAL P. crispum, PAL Petroselinum crispum (P24481); PAL P. luminescens, PAL Photorhabdus luminescens sp. laumondii TT01 (NP_929491); PAL S. maritimus, PAL Streptomyces maritimus (AAF81735); TAL C. pinensis, TAL Chitinophaga pinensis (YP_003121550); TAL R. sphaeroides, TAL Rhodobacter sphaeroides 2.4.1 (YP_355075); TAL S. espanaensis, TAL Saccharothrix espanaensis (ABC88669); TAL S. sp. Tü 4128, TAL Streptomyces sp. Tü 4128 (AEV23249). The selectivity switch reported by Watts et al. (2006) is boxed in motive 1. The residue at the first position (here residue 140) varied between Phe (F) and His (H) in PAL sequences, whereas TAL or PAL with TAL activity have a His (H) at the first position. Furthermore, a second conserved residue was reported (Berner et al., 2006), which is boxed in motive 2 (here residue 507) and was always Glu (E) for HAL and Gln (Q) for TAL or PAL. The alignment was performed using Geneious 6.1.7 ClustalW default settings. Coloured residues indicate a similarity ≥ 75%. Fig. S2. SDS-PAGE analysis of purified proteins. Left side: Purified FlxA (expected size 58 kDa). Right side: Purified FlxY (expected size 49 kDa). Marker: PageRuler™ Unstained protein ladder (Fermentas). Fig. S3. GC-MS analysis of FlxA enzyme assays. A. Mass spectrum of the product observed after incubation of FlxA with L-tyrosine (I) and 4-coumaric acid standard (II). B. Chromatogram of an assay containing FlxA and L-phenylalanine (I), the mass spectrum of the product at 6.1 min from the above chromatogram (II) and the mass spectrum of E-cinnamic [file mbt20007-0232-sd1.pdf]

Supplementary Information

Table S1: Strains and plasmids used in this work.

Strain or plasmid	description	Source or reference
strains		
E. coli DH10B	F– *mcr*A Δ(*mrr-hsd*RMS-*mcr*BC) Φ80*lac*ZΔM15 Δ*lac*X74 *rec*A1 *end*A1 *ara*D139 Δ(*ara leu*) 7697 *gal*U *gal*K *rps*L *nup*G λ–	(Grant *et al.*, 1990)
E. coli BL21 (DE3)	F– *omp*T *hsd*SB(rB–, mB–) *gal dcm rne*131 (DE3)	Invitrogen
C. pinensis DSM 2588	Wild type	DSMZ
CS1853	pCOLA-1853 in *E. coli* BL21 (DE3)	This work
CS1877	pCOLA-1877 in *E. coli* BL21 (DE3)	This work
plasmids		
pCOLADuet-1	Expressionplasmid, His_6-Tag, Km^R	Merck (Darmstadt)
pCOLA-1853	*flxA* (*cpin_1853*) in pCOLADuet-1, N-terminal His_6-tag, Km^R	This work
pCOLA-1877	*flxY* (*cpin_1877*) in pCOLADuet-1, N-terminal His_6-tag, Km^R	This Work

Table S2: Primers and PCR products used in this study.

Primers or PCR products	Nucleotide sequence (5´- 3´) or description*	Purpose
primers		
1853fw	TAGA<u>GGATCC</u>ATGGTTGTTTTAGGAAGTAAGGTGCTTTC	Amplification 1853fragment
1853rev	TTAT<u>CTCGAG</u>GCACATTTCATCCGTTAATTGTTTTAGTAGG	Amplification 1853fragment
1877fw	GCAA<u>GGATCC</u>GATGTACATACCCGACATTGAACTG	Amplification 1877fragment
1877rev	CGCC<u>AAGCTT</u>CTTCTCCTTCTGATTATCGTCTG	Amplification 1877fragment
PCR-products		
1853fragment	PCR-product of *cpin_1853* with *Bam*HI/*Xho*I restriction sites	Cloning into pCOLA-DUET1
1877fragment	PCR-product of *cpin_1877* with *Bam*HI/*Hin*dIII restriction sites	Cloning into pCOLA-DUET1

*recognition sites of restriction endocnucleases are underlined

Table S3: High-resolution MALDI-orbitrap-MS of CoA-thioesters from FlxY substrate assays.

Substrate	mass CoA-thioester [Da]	*m/z* calc.	*m/z* det.	error *m/z*	error ppm
Phenylacetic acid	885.1560	886.1633	n.d.		
E-3-Indoleacrylic acid	936.1669	937.1742	937.1769	0.0027	2.9
Indole-3-propionic acid	938.1825	939.1898	939.1911	0.0013	1.4
Isovaleric acid	851.1716	852.1789	n.d.		
3-Methylvaleric acid	865.1873	866.1946	n.d.		
3-Chlorocinnamic acid	931.1170	932.1243	932.1260	0.0017	1.9
3-Phenylpropionic acid	899.1716	900.1789	900.1793	0.0004	0.5
3-(4-Chlorophenyl)propionic acid	919.1170	920.1243	n.d.		
E-Cinnamic acid	897.1560	898.1633	898.1644	0.0011	1.2
Decanoic acid	921.2499	922.2572	n.d.		
4-Coumaric acid	913.1509	914.1582	914.1596	0.0015	1.6

n.d. = not detectable

Table S4. Predicted gene clusters for flexirubin biosynthesis in *C. pinensis*. Domain guided annotation is based on conserved domains detected by BLAST-P of *C. pinensis* DSM 2588 primary sequences against the genome of *F. johnsoniae* UW101.

**Name**	**Genelocus** [cpin_]	**NCBI annotation**	**domain guided annotation**	*C. pinensis*						*F. johnsoniae*					
				Max score	Total score	coverage [%]	E value	identity [%]	Accession number	Max score	Total score	coverage [%]	E value	identity [%]	Accession number
flxA	1853	histidine ammonia-lyase	ammonia-lyase	1061	1061	100	0	100	YP_003121550.1	640	640	97	0	60	YP_001193461.1
flxB	1854	short-chain dehydrogenase/reductase SDR		484	484	100	7.00E-175	100	YP_003121551.1	303	303	100	3.00E-104	60	YP_001193459.1
flxC	1855	beta-ketoacyl synthase	KAS I or II	838	838	100	0	100	YP_003121552.1	580	580	99	0	67	YP_001193458.1
flxD	1856	phosphopantetheine-binding protein	ACP	169	169	100	1.00E-55	100	YP_003121553.1	93.6	93.6	96	1.00E-26	59	YP_001193457.1
flxE	1857	lipid A biosynthesis acyltransferase	acyltransferase	615	615	100	0	100	YP_003121554.1	293	293	98	1.00E-98	48	YP_001193456.1
flxF	1858	hypothetical protein	dehydratase-like	281	281	100	4.00E-98	100	YP_003121555.1	76.6	76.6	88	7.00E-19	32	YP_001193453.1
flxG	1859	hypothetical protein		477	477	100	4.00E-172	100	YP_003121556.1						
flxH	1860	thioesterase superfamily protein	thioesterase	309	309	100	7.00E-109	100	YP_003121557.1	152	152	90	8.00E-48	55	YP_001193446.1
flxI	1861	beta-ketoacyl synthase	KAS I or II	787	787	100	0	100	YP_003121558.1	328	328	100	1.00E-111	43	YP_001193445.1
flxJ	1862	type 12 methyltransferase	methyltransferase	737	737	100	0	100	YP_003121559.1						
flxK	1863	tryptophan halogenase	halogenase	855	855	100	0	100	YP_003121560.1	379	379	98	7.00E-129	45	YP_001193462.1
flxL	1864	hypothetical protein	condensing enzyme	424	424	100	3.00E-152	100	YP_003121561.1	152	152	92	3.00E-46	41	YP_001193442.1
flxM	1865	phosphopantetheine-binding protein	ACP	173	173	100	3.00E-57	100	YP_003121562.1	95.1	95.1	97	3.00E-27	51	YP_001193441.1
flxN	1866	beta-ketoacyl synthase	KAS I or II	843	843	100	0	100	YP_003121563.1	370	370	99	1.00E-125	46	YP_001193440.1
flxO	1867	beta-ketoacyl synthase	condensing enzyme	738	738	100	0	100	YP_003121564.1	251	251	98	2.00E-80	37	YP_001193439.1
flxP	1868	polysaccharide deacetylase	PS-deacetylase	530	530	100	0	100	YP_003121565.1	194	194	90	3.00E-61	37	YP_001193438.1
flxQ	1869	outer membrane lipoprotein carrier protein LolA	Lipoprotein-Carrier	435	435	100	1.00E-156	100	YP_003121566.1	93.6	93.6	91	6.00E-24	29	YP_001193437.1
flxR	1870	hypothetical protein		427	427	100	1.00E-153	100	YP_003121567.1	79.7	79.7	96	5.00E-19	30	YP_001193436.1
flxS	1871	hypothetical protein	dehydratase-like	254	254	100	7.00E-88	100	YP_003121568.1	75.9	75.9	96	7.00E-19	29	YP_001193433.1
flxT	1872	family 2 glycosyl transferase	glycosyltransferase	832	832	100	0	100	YP_003121569.1	406	406	93	9.00E-140	53	YP_001193431.1
flxU	1873	phospholipid/glycerol acyltransferase	contains acyl- and methyltransferase domains, predicted exporter	2659	2659	100	0	100	YP_003121570.1	851	851	91	0	37	YP_001193430.1
flxV	1874	all-trans-retinol 13,14-reductase	reductase	1049	1049	100	0	100	YP_003121571.1	495	495	99	2.00E-171	47	YP_001193429.1
flxW	1875	peptidase C45 acyl-coenzyme A:6- aminopenicillanic acid acyl-transferase	Ntn_hydrolase	1173	1173	100	0	100	YP_003121572.1	454	454	88	7.00E-154	46	YP_001193428.1
flxX	1876	holo-ACP synthase	ACP-synthase	256	256	100	4.00E-91	100	YP_003121573.1						
flxY	1877	coenzyme F390 synthetase-like protein	CoA-ligase	898	898	100	0	100	YP_003121574.1	496	496	99	3.00E-174	52	YP_001193427.1
darE	6843	hypothetical protein	N-terminal Beta-ketoacyl synthase domain	741	741	100	0	100	YP_003126445.1	244	244	98	4.00E-78	39	YP_001193439.1
darD	6844	beta-ketoacyl synthase	KAS I or II	833	833	100	0	100	YP_003126446.1	394	394	99	5.00E-135	49	YP_001193440.1
darC	6845	acyl-carrier protein	ACP	169	169	100	2.00E-55	100	YP_003126447.1	102	102	97	5.00E-30	58	YP_001193441.1
darJ	6846	ABC transporter	ABC-Transporter/Permease	872	872	100	0	100	YP_003126448.1	296	296	99	3.00E-96	37	YP_001193447.1
darI	6847	ABC transporter	ABC-Transporter/ATPase	501	501	100	0	100	YP_003126449.1	248	248	99	2.00E-82	53	YP_001193448.1
darH	6848	hypothetical protein	BtrH-like peptidase	705	705	100	0	100	YP_003126450.1	375	375	95	2.00E-129	53	YP_001193449.1
darG	6849	hypothetical protein	chulike-protein	275	275	100	4.00E-96	100	YP_003126451.1	151	151	98	6.00E-48	50	YP_001193450.1
darB	6850	3-oxoacyl-ACP synthase	DAR-Cyclase	790	790	100	0	100	YP_003126452.1	436	436	100	6.00E-152	54	YP_001193454.1
darA	6851	hypothetical protein	DAR-Aromatase	620	620	100	0	100	YP_003126453.1	214	214	97	8.00E-68	35	YP_001193455.1
darF	6852	type 11 methyltransferase		439	439	100	7e-155	100	YP_003126454.1	84.7	84.7	47	2e-21	38	YP_001193863.1

Table S5. Gene cluster for flexirubin biosynthesis in *F. johnsoniae* UW101. Domain guided annotation is based on conserved domains detected by BLAST-P.

**Genelocus** [Fjoh_]	**NCBI annotation**	**domain guided annotation**
1074	nifR3 family TIM-barrel protein	
1075	phenylacetate-CoA ligase	CoA-ligase
1076	peptidase C45, acyl-coenzyme A:6-aminopenicillanic acid acyl-transferase	Ntn-hydrolase
1077	all-trans-retinol 13,14-reductase	reductase
1078	phospholipid/glycerol acyltransferase	predicted exporter- and acyltransferase domain
1079	glycosyl transferase family protein	glycosyltransferase
1080	hypothetical protein	
1081	beta-hydroxyacyl-(acyl-carrier-protein) dehydratase, FabA/FabZ	dehydratase
1082	hypothetical protein	contains outer membrane protein beta-barrel domain
1083	hypothetical protein	contains outer membrane protein beta-barrel domain
1084	hypothetical protein	conserved hypothetical protein
1085	outer membrane lipoprotein carrier protein LolA	Lipoprotein-carrier
1086	polysaccharide deacetylase	PS-deacetylase
1087	3-oxoacyl-ACP synthase	condensing enzyme
1088	beta-ketoacyl synthase	KAS I or II
1089	hypothetical protein	ACP
1090	hypothetical protein	condensing enzyme
1091	virulence protein-like protein	Virulence protein RhuM family
1092	hypothetical protein	
1093	beta-ketoacyl synthase	KAS I or II
1094	thioesterase superfamily protein	thioesterase
1095	ABC-2 type transporter	ABC-transporter
1096	ABC transporter-like protein	ABC-transporter
1097	hypothetical protein	predicted NlpC/p60-like peptidase
1098	hypothetical protein	conserved hypothetical protein
1099	hypothetical protein	
1100	hypothetical protein	
1101	hypothetical protein	dehydratase or thioesterase
1102	3-oxoacyl-ACP synthase	DAR-cyclase DarB/KAS III
1103	hypothetical protein	DAR-aromatase DarA
1104	lipid A biosynthesis acyltransferase	acyltransferase
1105	hypothetical protein	ACP
1106	beta-ketoacyl synthase	KAS I or II
1107	3-oxoacyl-ACP synthase	DAR-cyclase
1108	dialkylrecorsinol condensing protein	DAR-aromatase
1109	histidine ammonia-lyase	ammonia-lyase
1110	tryptophan halogenase	halogenase

Table S6: Comparison of the enzymatic properties of bacterial 4CLs (4-coumarate-CoA ligase) and CCLs (cinnamate-CoA ligase). Values were obtained from assays with 4-coumarate (4CA) and *E*-cinnamate (CA) as substrates.

**Enzyme**	**Organism**	**K_M [µM]**		**v_{max} [nM s^{-1}]**		**k_{cat}/K_M [M^{-1} s^{-1}]**	
		4CA	CA	4CA	CA	4CA	CA
FlxY	*Chitinophaga pinensis*	17	868	444	190	52977	437
Unknown CCL[a]	*Pseudomonas putida*	16	252	4.83	0.34	nd	nd
ScCCL[b]	*Streptomyces coelicolor* A3(2)	131	190	nd	nd	1545	2499

nd = not determined

[a] values taken from Zenk *et al*., 1980

[b] values taken from Kaneko *et al*., 2003

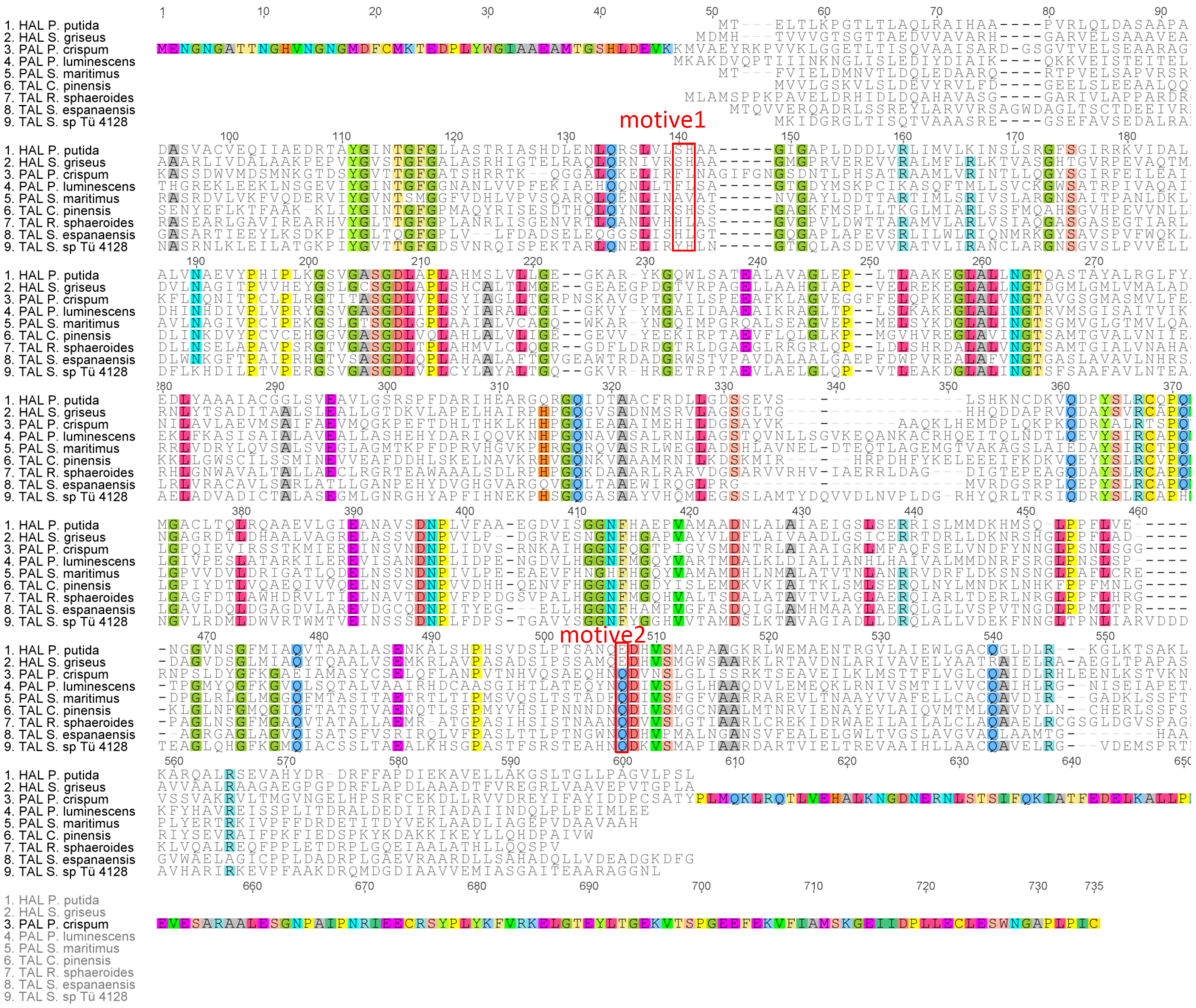

Figure S1. ClustalW multiple alignment of primary sequences of FlxA from *C. pinensis* with those of histidine ammonia-lyases (HAL), phenylalanine ammonia-lyases (PAL) and tyrosine ammonia-lyases (TAL) with known substrate specificity. Sequences used for the alignment were abbreviated as follows (with GenBank accession numbers in parentheses): HAL P. putida, HAL *Pseudomonas putida* (P21310); HAL S. griseus, HAL *Streptomyces griseus* (AAA26769); PAL P. crispum, PAL *Petroselinum crispum* (P24481); PAL P. luminescens, PAL *Photorhabdus luminescens* subsp. *laumondii* TT01 (NP_929491); PAL S. maritimus, PAL *Streptomyces maritimus* (AAF81735); TAL C. pinensis, TAL *Chitinophaga pinensis* (YP_003121550); TAL R. sphaeroides, TAL *Rhodobacter sphaeroides* 2.4.1 (YP_355075); TAL S. espanaensis, TAL *Saccharothrix espanaensis* (ABC88669); TAL S. sp Tü 4128, TAL *Streptomyces* sp. Tü 4128 (AEV23249). The selectivity switch reported by Watts et al., 2006 is boxed in motive 1. The residue at the first position (here residue 140) varied between Phe (F) and His (H) in PAL sequences, whereas TAL or PAL with TAL activity have a His (H) at the first position. Furthermore a second conserved residue was reported (Berner *et al.*, 2006) which is boxed in motive 2 (here residue 507) and was always Glu (E) for HAL and Gln (Q) for TAL or PAL. The alignment was performed using Geneious 6.1.7 ClustalW default settings. Colored residues indicate a similarity ≥ 75%.

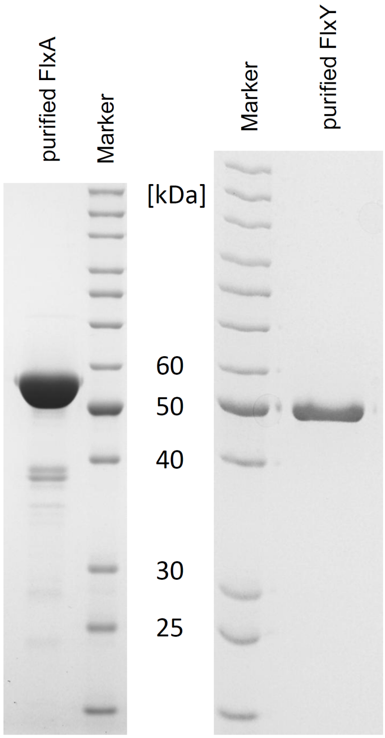

Figure S2. SDS-PAGE analysis of purified proteins. Left side purified FlxA (expected size 58 kDa). Right side purified FlxY (expected size 49 kDa). Marker: PageRuler™ Unstained protein ladder (Fermentas).

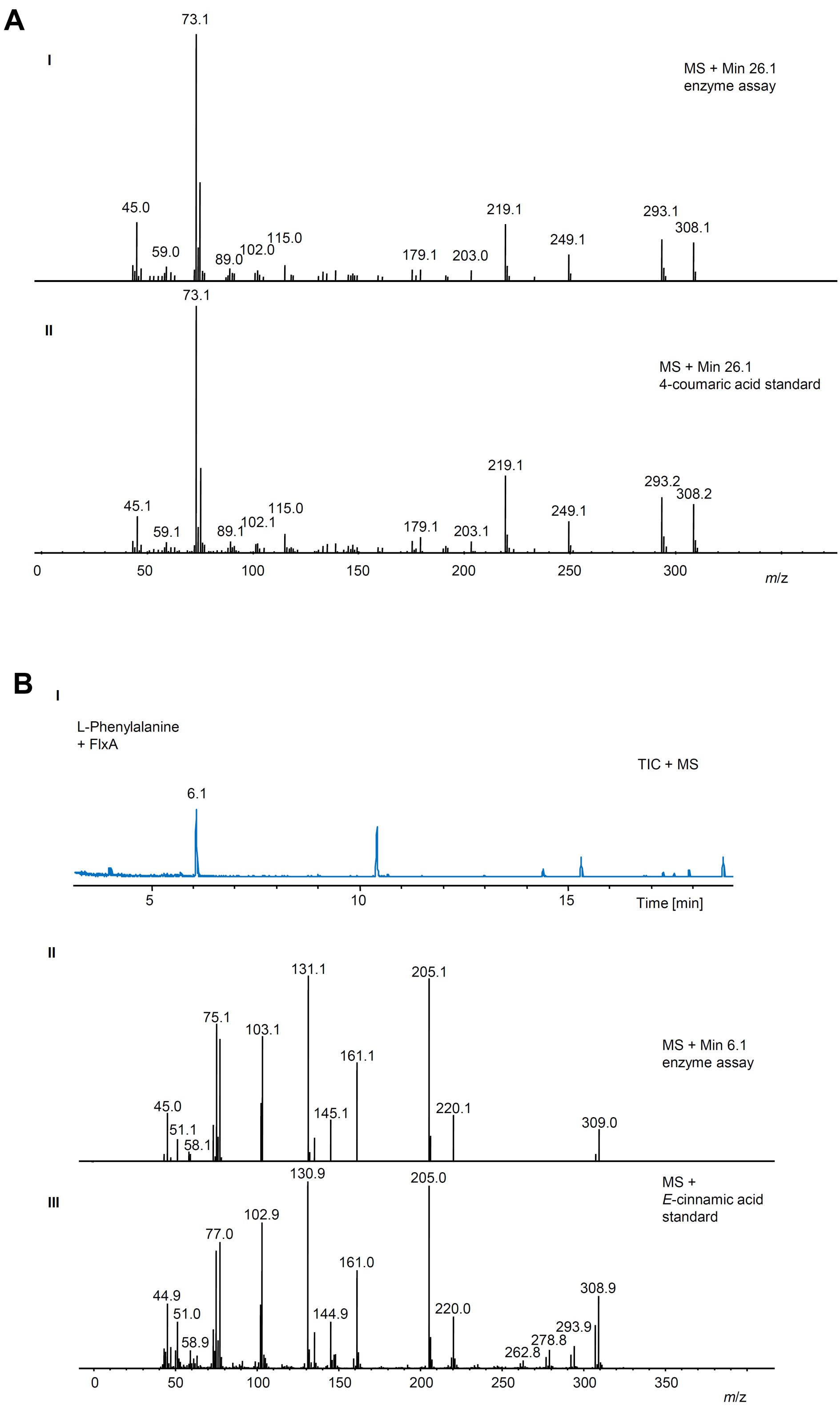

Figure S3. GC-MS analysis of FlxA enzyme assays. **A**: mass spectrum of the product observed after incubation of FlxA with L-tyrosine (I) and 4-coumaric acid standard (II). **B:** Chromatogram of an assay containing FlxA and L-phenylalanine (I), the mass spectrum of the product at 6.1 min from the above chromatogram (II) and the mass spectrum of *E*-cinnamic acid standard (III).

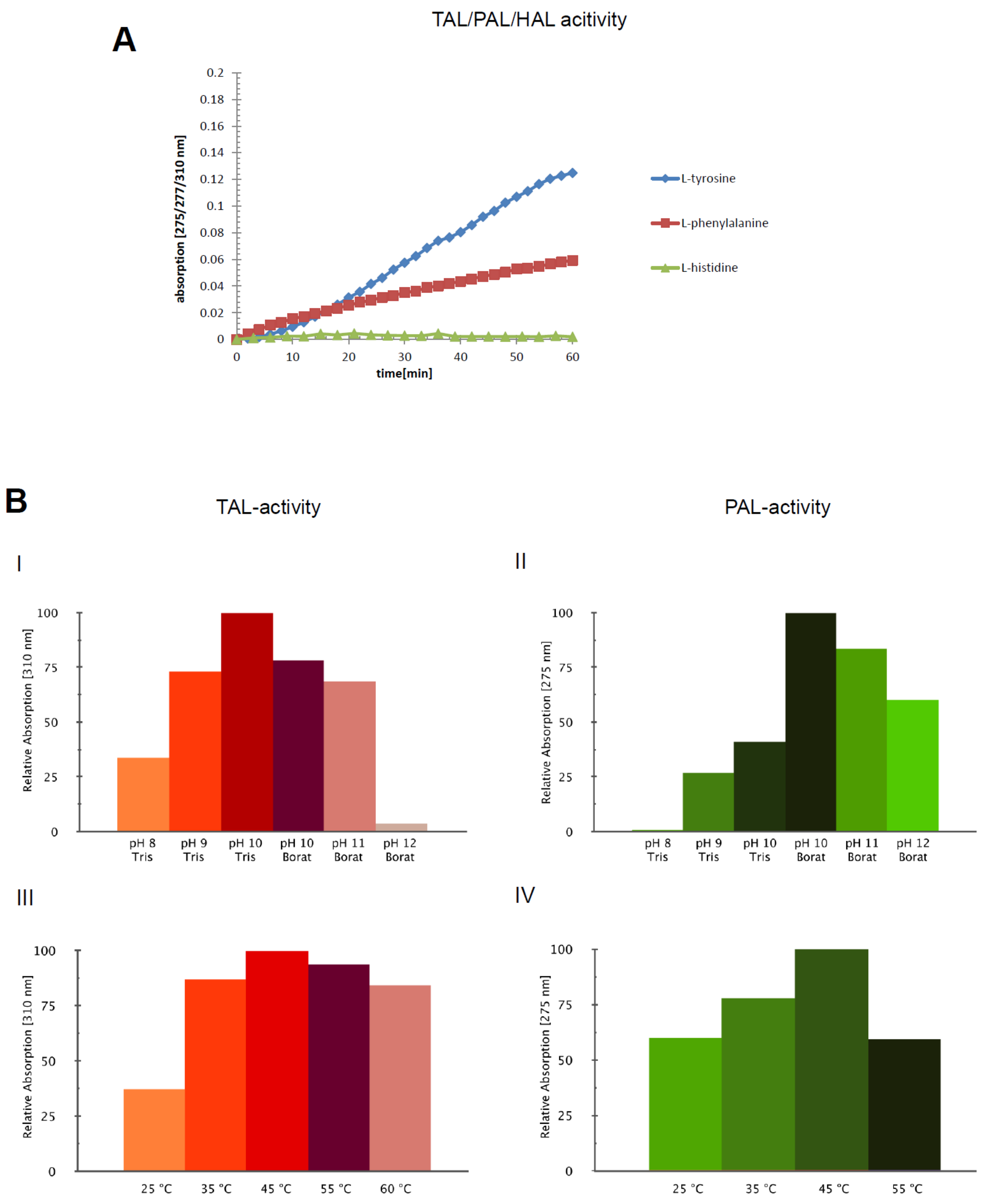

Figure S4. Photometric FlxA enzyme assays. **A**: Endpoint assay detecting the enzymatic conversion of L-tyrosine (blue), L-phenylalanine (red) and L-histidine (green) by measuring the absorption increase of 4-coumaric acid (310 nm), *E*-cinnamic acid (275 nm) or urocanic acid (277 nm), respectively. **B:** pH-dependence of FlxA activity with L-tyrosine (I) and L-phenylalanine (II). temperature-dependence of FlxA activity with L-tyrosine (III) and L-phenylalanine (IV). TAL: tyrosine ammonia-lyase; PAL: phenylalanine ammonia-lyase; HAL: histidine ammonia-lyase.

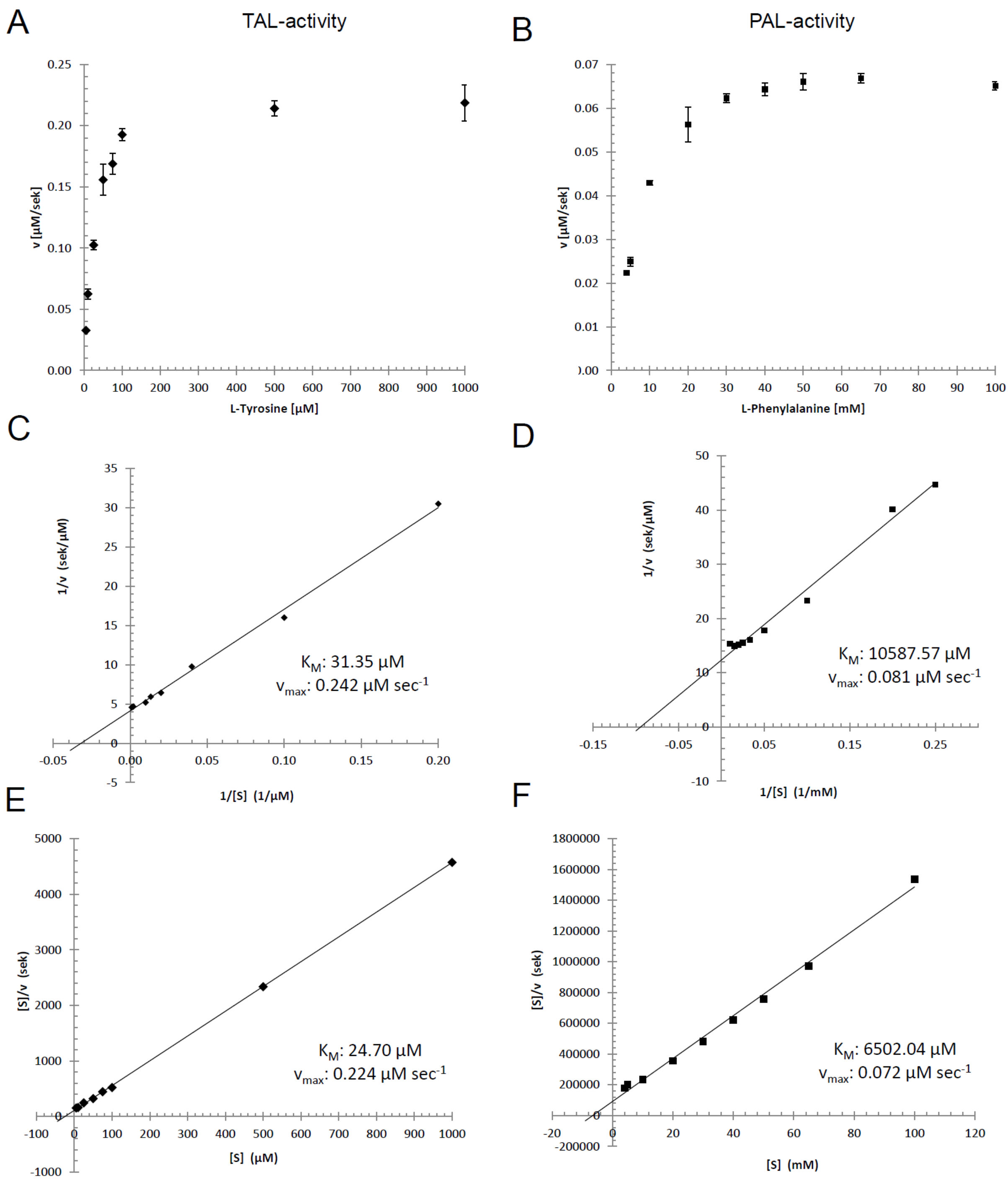

Figure S5. Michaelis-Menten kinetics of FlxA. Substrate saturation plots of FlxA with L-tyrosine (**A**) and L-phenylalanine (**B**) and their respective linearization as Lineweaver-Burk (**C** and **D**) or Hanes-Woolf plots(**E** and **D**). TAL: tyrosine ammonia-lyase; PAL: phenylalanine ammonia-lyase.

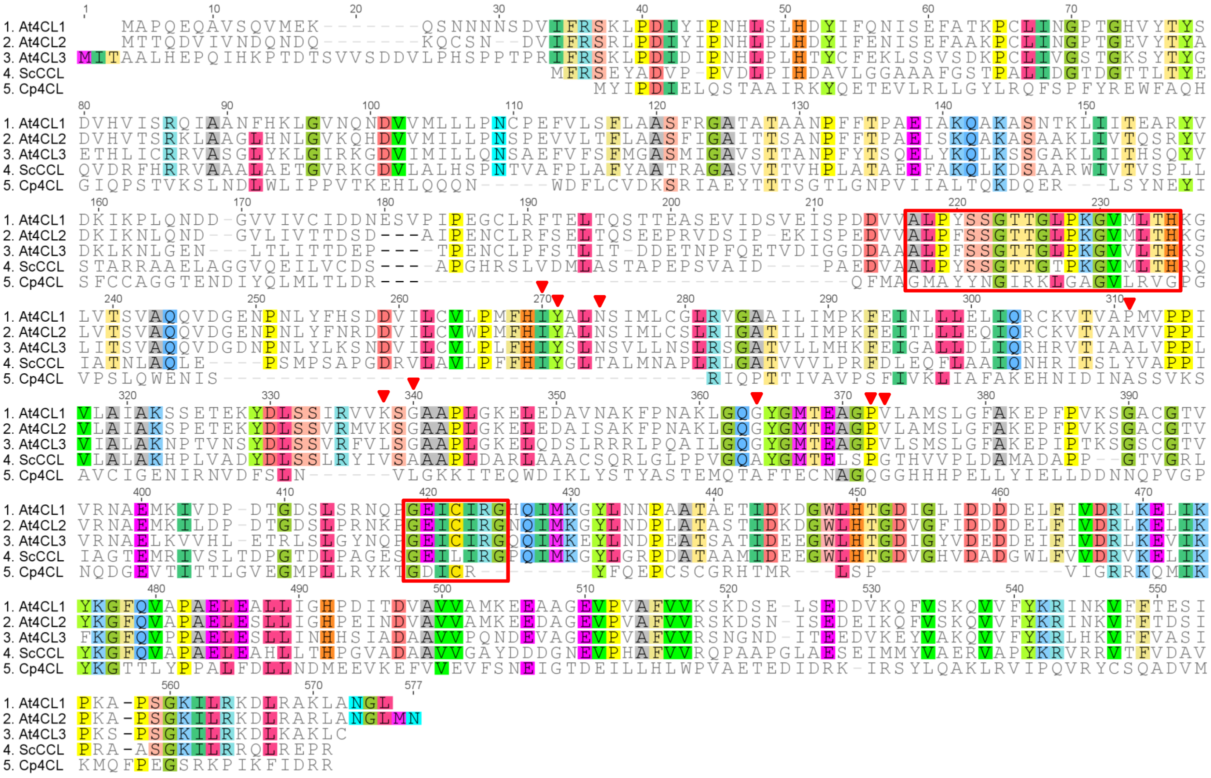

Figure S6. ClustalW multiple alignment of primary sequences of FlxY from *Chitinophaga pinensis* with three 4-coumarate-CoA ligases (4CL) from *Arabidopsis thaliana* and the *E*-cinnamate-CoA ligase (CCL) ScCCL from *Streptomyces coelicolor* A3(2)*.* Sequences used for the alignment were abbreviated as follows (with GenBank accession numbers in parentheses): At4CL1, *A. thaliana* 4CL1 (AAA82888.1); At4CL2, *A. thaliana* 4CL2 (AF106086_1); At4CL3, *A. thaliana* 4CL3 (AF106088_1); ScCCL, *S. coelicolor* A3 (2) CCL (CAB95894); Cp4CL, *C. pinensis* 4CL (YP_003121574.1). Two conserved peptide boxes from 4CL are boxed in red and nine residues which are suggested to form the At4CL substrate binding pocket are marked by red triangles (Kaneko *et al.*, 2003). The alignment was performed using Geneious 6.1.7 ClustalW default settings. Colored residues indicate a similarity ≥ 75%.

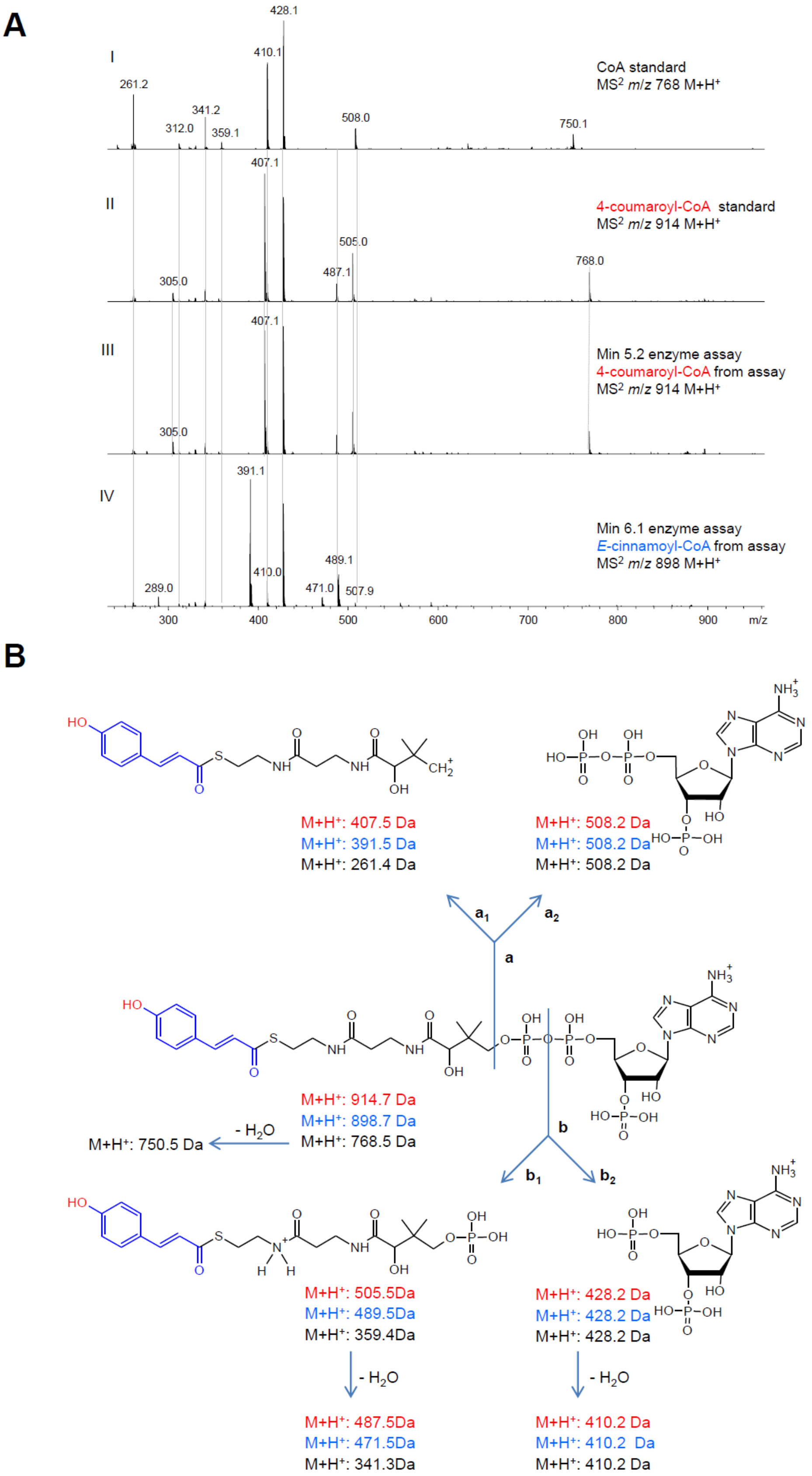

Figure S7. HPLC-MS analysis of enzyme assays with FlxY. **A**: Mass spectra of CoA (I), 4-coumaroyl-CoA standard (II) and products detected in enzyme assays containing FlxY and 4-coumaric acid (III) or *E*-cinnamic acid (IV) as substrates. **B**: theoretical fragmentation tree explaining the above mass spectra with randomly positioned charges.

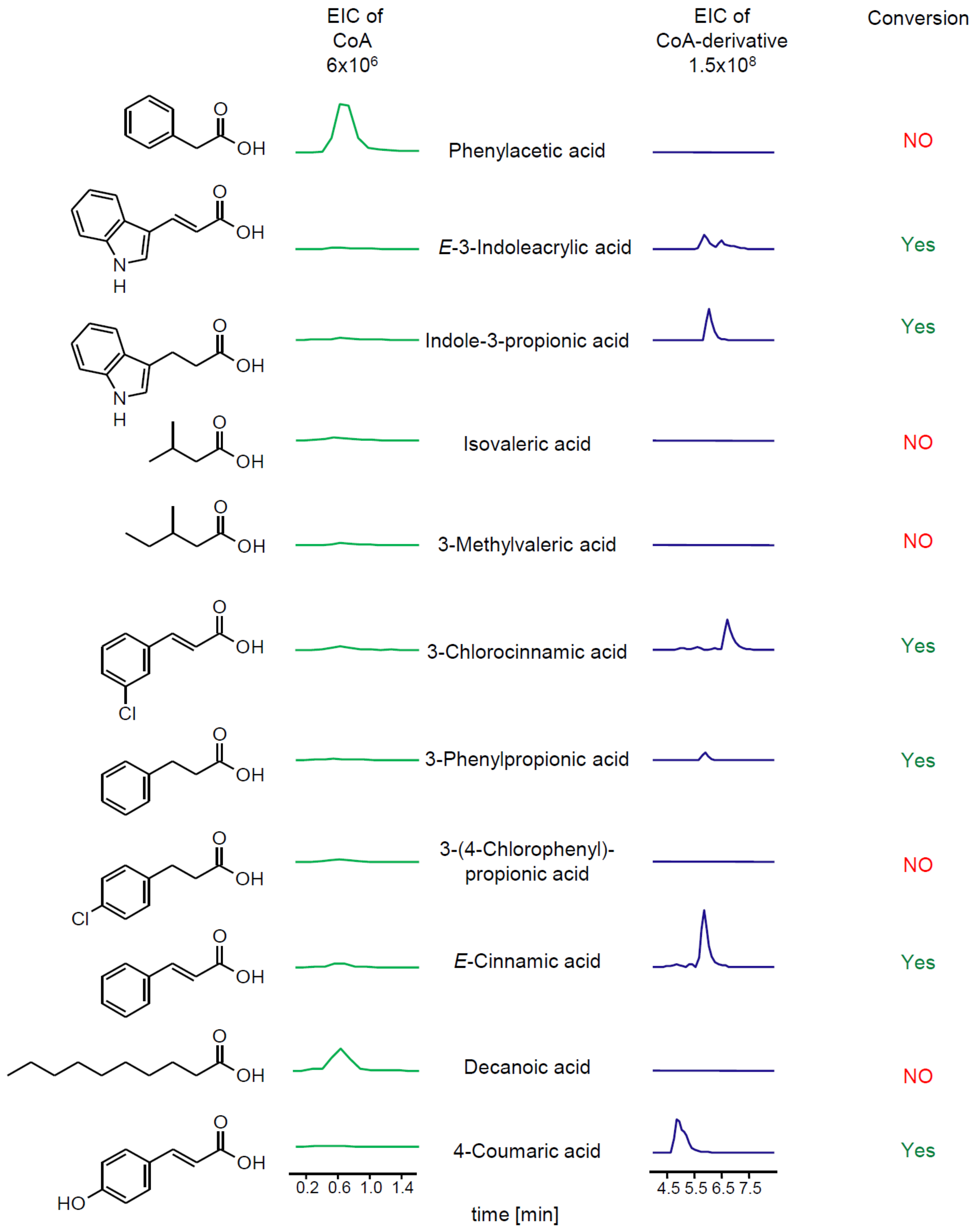

Figure S8. HPLC-MS analysis of the substrate specificity of FlxY. The structures of the substrates, the EIC from CoA (green) and the EIC of expected substrate-CoA thioesters (blue) are shown.

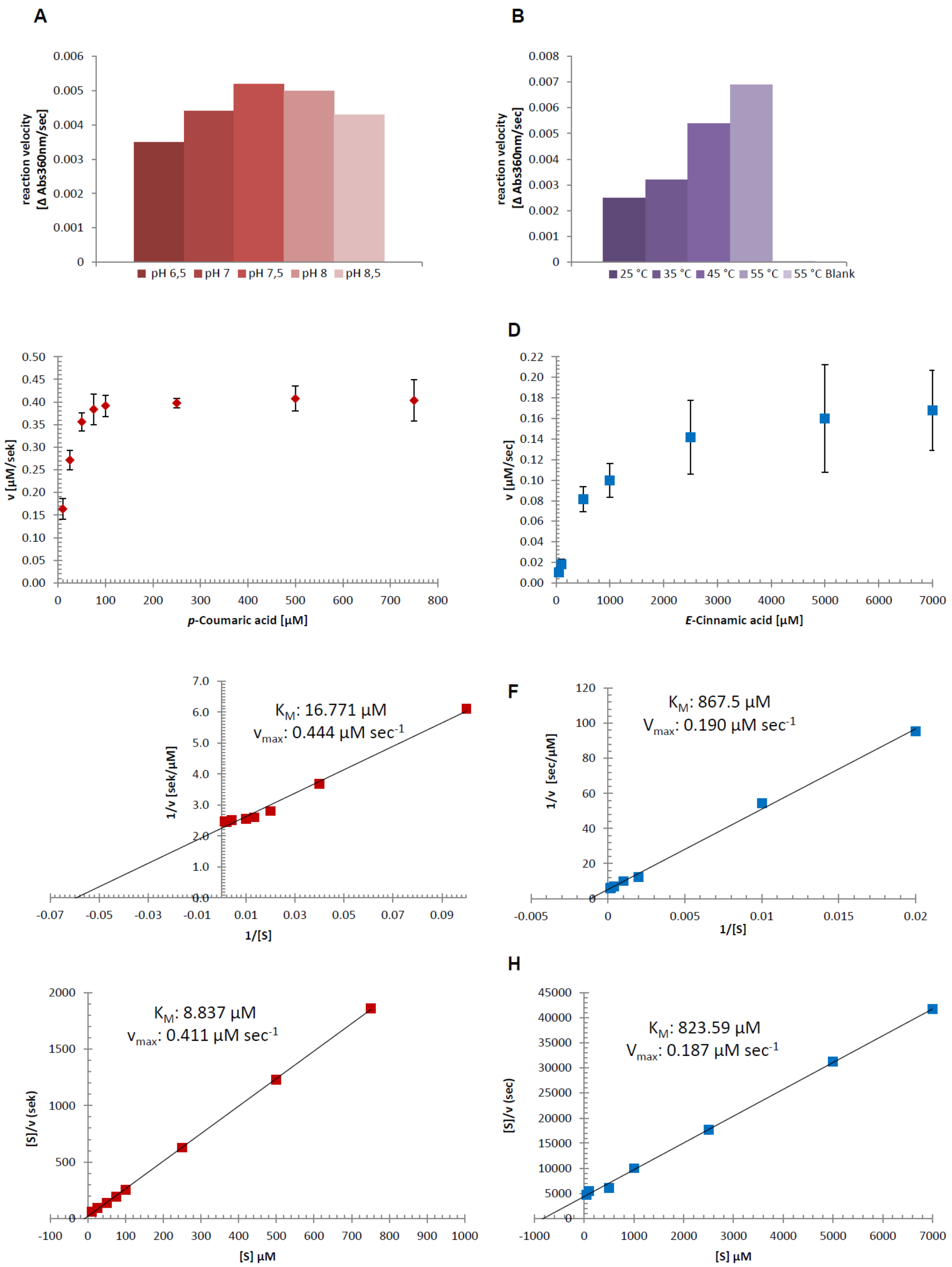

Figure S9. Results of enzyme assays with FlxY. pH (A) and temperature optimum (B) of the coupled enzyme assay. Substrate saturation plots of FlxY with 4-coumaric acid (**C**) and *E*-cinnamic acid (**D**) and their respective linearization as Lineweaver-Burk (**E** and **F**) or Hanes-Woolf plots (**G** and **H**).

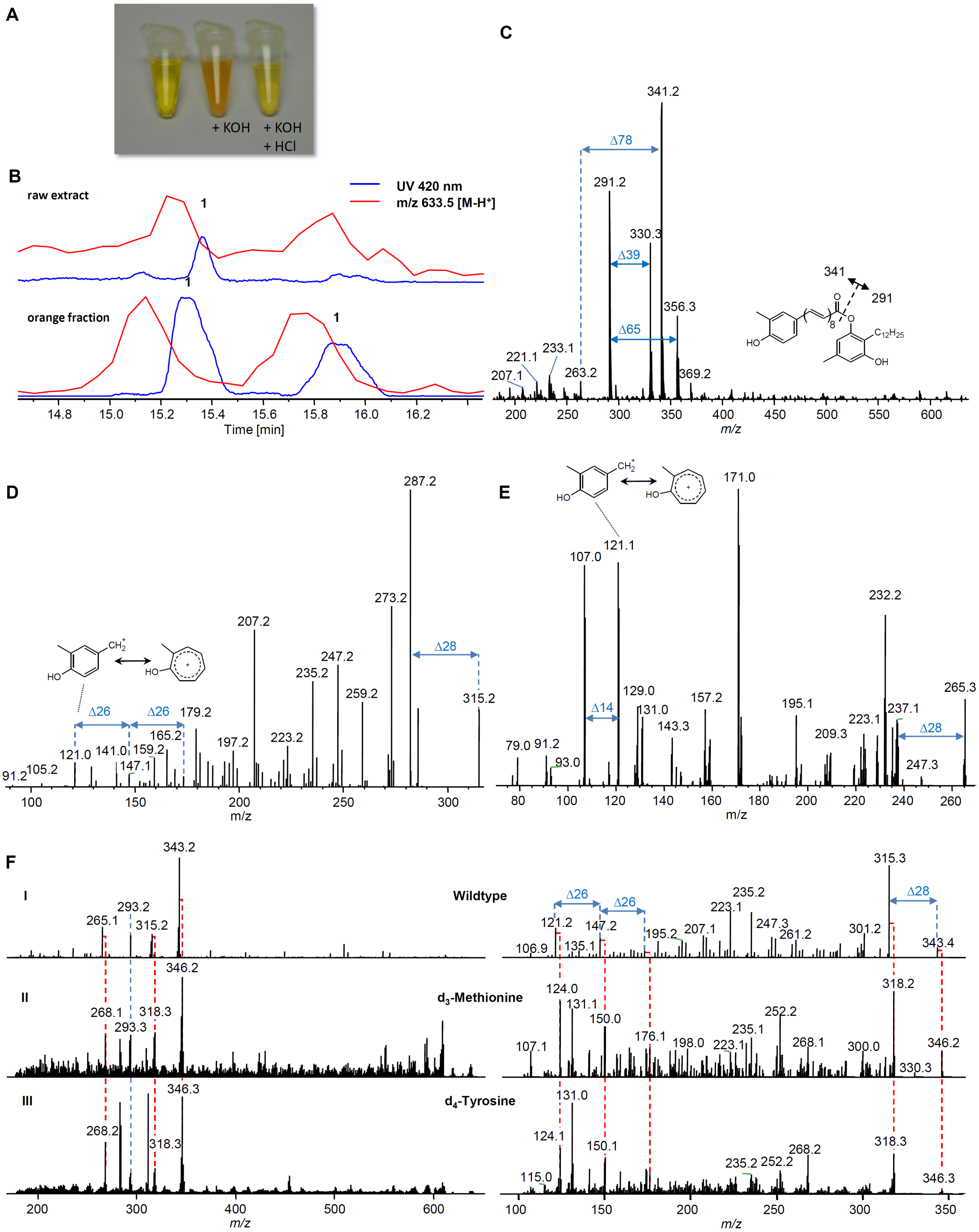

Figure S10. KOH-test, mass spectra and HPLC-traces from *C. pinensis* flexirubin **1**. **A**: Crude acetone extract of *C. pinensis* culture (left) showing the reversible colour shift after addition of base (middle) or base and acid (right). **B:** HPLC-MS-chromatograms of *C. pinensis*-extract before (upper trace) and after fractionation by column chromatography (lower trace). UV at 420 nm (blue line) and an EIC *m/z* 633.5 $[M\text{-}H^+]$ are shown. The occurrence of an additional signal after purification may be explained by isomerisation. Chromatograms are drawn to the same scale. **C**: HPLC-ESI-MS^2 of **1** *m/z* 633.5 $[M\text{-}H^+]$. MALDI-iontrap-MS^3 spectra of *m/z* 315.2 (**D**) and *m/z* 265.3 (**E**) with *m/z* 634.4 $[M]^{+\cdot}$ as precursor. **F**: On the left side MALDI-iontrap-MS^2 of wildtype **1** *m/z* 634.4 $[M]^{+\cdot}$ (I) and *m/z* 637.4 $[M]^{+\cdot}$ from feeding experiments with d_3-methionine (II) and d_4-tyrosine (III). The resulting fragments *m/z* 343.2 for wildtype and *m/z* 346.2 or *m/z* 346.3 for the feeding experiments, were further fragmented and the corresponding MS^3 mass spectra are depicted on the right side.

References

Berner, M., Krug, D., Bihlmaier, C., Vente, A., Müller, R. and Bechthold, A. (2006) Genes and Enzymes Involved in Caffeic Acid Biosynthesis in the Actinomycete *Saccharothrix espanaensis*. J Bacteriol 188: 2666-2673

Grant, S.G.N., Jessee, J., Bloom, F.R. and Hanahan, D. (1990) Differential Plasmid Rescue From Transgenic Mouse DNAs Into *Escherichia coli* Methylation-Restriction Mutants. Proc Natl Acad Sci USA 87: 4645-4649.

Kaneko, M., Ohnishi, Y. and Horinouchi, S. (2003) Cinnamate:Coenzyme A Ligase from the Filamentous Bacterium *Streptomyces coelicolor* A3(2). J Bacteriol 185: 20-27.

Watts, K.T., Mijts, B.N., Lee, P.C., Manning, A.J. and Schmidt-Dannert, C. (2006) Discovery of a substrate selectivity switch in tyrosine ammonia-lyase, a member of the aromatic amino acid lyase family. Chem Biol 13: 1317-1326.

Zenk, M.H., Ulbrich, B., Busse, J. and Stöckigt, J. (1980) Procedure for the enzymatic synthesis and isolation of cinnamoyl-CoA thiolesters using a bacterial system. Anal Biochem 101: 182-187.
